# Impact of the Dual Antiplatelet Therapy Score on Clinical Outcomes in Acute Coronary Syndrome Patients Receiving P2Y12 Inhibitor Monotherapy

**DOI:** 10.3389/fcvm.2021.772820

**Published:** 2022-02-24

**Authors:** Sheng-Wei Huang, Po-Wei Chen, Wen-Han Feng, I-Chang Hsieh, Ming-Yun Ho, Chung-Wei Cheng, Hung-I Yeh, Ching-Pei Chen, Wei-Chun Huang, Ching-Chang Fang, Hui-Wen Lin, Sheng-Hsiang Lin, Chin-Feng Tsai, Chun-Hung Su, Yi-Heng Li

**Affiliations:** ^1^School of Medicine, Chung Shan Medical University Hospital, Chung Shan Medical University, Taichung, Taiwan; ^2^College of Medicine, National Cheng Kung University Hospital, National Cheng Kung University, Tainan, Taiwan; ^3^Kaohsiung Municipal Ta-Tung Hospital and Kaohsiung Medical University Hospital, Kaohsiung, Taiwan; ^4^Chang Gung Memorial Hospital, Chang Gung University College of Medicine, Taoyuan, Taiwan; ^5^Mackay Memorial Hospital, Taipei, Taiwan; ^6^Changhua Christian Hospital, Changhua, Taiwan; ^7^Kaohsiung Veterans General Hospital, Fooyin University, Kaohsiung and National Yang Ming University, Taipei, Taiwan; ^8^Tainan Municipal Hospital, Tainan, Taiwan; ^9^College of Medicine, Institute of Clinical Medicine, National Cheng Kung University, Tainan, Taiwan; ^10^Department of Public Health, College of Medicine, National Cheng Kung University, Tainan, Taiwan; ^11^Biostatistics Consulting Center, College of Medicine, National Cheng Kung University Hospital, National Cheng Kung University, Tainan, Taiwan

**Keywords:** P2Y12 inhibitor, acute coronary syndrome, DAPT score, P2Y12 inhibitor monotherapy, clinical outcome

## Abstract

**Background:**

Dual antiplatelet therapy (DAPT) score is used to stratify ischemic and bleeding risk for antiplatelet therapy after percutaneous coronary intervention (PCI). This study assessed the association between the DAPT score and clinical outcomes in acute coronary syndrome (ACS) patients who were treated with P2Y12 inhibitor monotherapy.

**Methods:**

A total of 498 ACS patients, with early aspirin discontinuation for various reasons and who received P2Y12 inhibitor monotherapy after PCI, were enrolled during the period from January 1, 2014 to December 31, 2018. The efficacy and safety between those with low (<2) and high (≥2) DAPT scores were compared during a 12-month follow-up after PCI. Inverse probability of treatment weighting was used to balance the covariates between the two groups. The primary endpoint was a composite outcome of all-cause mortality, recurrent ACS or unplanned revascularization, and stroke within 12 months. The safety endpoint was major bleeding, defined as Bleeding Academic Research Consortium (BARC) 3 or 5 bleeding.

**Results:**

The primary composite endpoint occurred in 11.56 and 14.38% of the low and high DAPT score groups, respectively. Although there was no significant difference in the primary composite endpoint between the two groups in the multivariate Cox proportional hazards models, the risk of recurrent ACS or unplanned revascularization was significantly higher in the high DAPT score group (adjusted hazard ratio [HR]: 1.900, 95% confidence interval [CI]: 1.095–3.295). The safety outcome for BARC 3 or 5 bleeding was similar between the two groups.

**Conclusions:**

Our results indicate that ACS patients receiving P2Y12 monotherapy with high DAPT score had an increased risk of recurrent ACS or unplanned revascularization.

## Introduction

Dual antiplatelet therapy (DAPT) with aspirin and P2Y12 inhibitor is the foundation therapy for acute coronary syndrome (ACS). Current guidelines recommend 12-month DAPT for patients with ACS who have received percutaneous coronary intervention (PCI) (1–3). However, DAPT-associated bleeding has raised concerns because bleeding complications increase the risk of morbidity and mortality (4–6). As patient-tailored antiplatelet therapy has become necessary, the DAPT score was developed to help physicians select patients who would benefit the most from longer or shorter DAPT after PCI (7). The DAPT scoring system includes eight positive predictors (smoking, diabetes, myocardial infarction [MI] at presentation, prior PCI or MI, paclitaxel-eluting stent, stent diameter <3 mm, congestive heart failure or left ventricular ejection fraction <30%, and vein graft stent) and one negative predictor (age) (7). For patients with a high DAPT score (≥2), who had an increased ischemic risk, treatment with extended DAPT beyond 1 year resulted in a reduction in ischemic events but without an increase in bleeding (7). The DAPT score has been validated as useful for stratifying ischemia and bleeding risk in other patient groups, including Asian patients, outside the derivation cohort of the DAPT trial (8–10). Although there were some controversial results regarding its discriminating ability in one study (11), the DAPT score seems to be a clinically useful tool for determining DAPT duration after PCI.

Recently, several randomized trials evaluated the efficacy and safety of very short duration DAPT (1–3 months) followed by P2Y12 inhibitor monotherapy in patients who underwent PCI for stable coronary artery disease (CAD) and/or ACS (12–16). The rationale for using a very short period of aspirin therapy is that the benefits of intensive antiplatelet therapy with DAPT generally outweigh the risk of bleeding in the first few weeks after ACS or PCI, when the thrombogenic potential is still high. However, this benefit dissipates over time after that period and the antiplatelet potency could be enough with P2Y12 inhibitor monotherapy during later periods (6, 17). Overall, these clinical trials demonstrated a significant reduction in bleeding with P2Y12 inhibitor monotherapy vs. standard 12-month DAPT but no significant difference in terms of major adverse cardiovascular events (MACEs) (18). Among these clinical trials, TICO study was the first performed in Asia, which compared ticagrelor monotherapy after 3 months of DAPT vs. standard 12-month DAPT in ACS patients undergoing PCI (16). The risk of major bleeding was decreased in the ticagrelor monotherapy group but the rate of MACEs was similar to standard DAPT. Since Asian patients carry a higher bleeding risk with DAPT (19), very short DAPT followed by P2Y12 inhibitor monotherapy may be an alternative choice for Asian ACS patients.

In the TICO study, there was a significant interaction between P2Y12 monotherapy vs. standard DAPT and the presence of multivessel disease for the primary outcome (16). In the *post-hoc* analysis of patients with ST elevation MI in the TICO trial, the incidence of MACEs was slightly higher in the ticagrelor monotherapy group compared with 12-month DAPT in those who underwent complex PCI (4.9 vs. 2.7%) (17). Although P2Y12 inhibitor monotherapy was recommended as an optional antiplatelet strategy with standard DAPT in the 2020 European Society of Cardiology Guidelines for the management of ACS (1), there is no useful clinical outcome-predictive tool for choosing between different strategies. The efficacy of P2Y12 inhibitor monotherapy in high risk ischemic patients is also unknown. The aim of this study was to evaluate the association between DAPT score and clinical outcomes in a cohort of ACS patients undergoing PCI and who received P2Y12 inhibitor monotherapy.

## Methods

### Study Population

This was a multicenter, retrospective, registration study and the detailed study design was published previously (20). In brief, we retrospectively recruited ACS patients who were admitted to the 8 major teaching hospitals in Taiwan from January 2014 to December 2018. Patients were eligible if they were ≥18 years old, were admitted with a major diagnosis of ACS, including ST elevation MI, non-ST elevation MI or unstable angina, received PCI with a bare metal stent (BMS) and/or contemporary drug-eluting stent (DES) implantation during hospitalization, survived to discharge, regularly followed up in an outpatient clinic for at least 1 year after discharge, and aspirin discontinuation within 6 months. We only studied a subset of ACS patients in whom aspirin was discontinued prematurely. In all enrolled patients, aspirin was prematurely discontinued within 6 months after PCI at the physician's discretion for different reasons. P2Y12 inhibitor monotherapy was used thereafter in all patients with either clopidogrel 75 mg daily or ticagrelor 90 mg twice daily. Prasugrel was not available during the study period in Taiwan. The exclusion criteria were patients with (1) a life-threatening malignancy with a life expectancy of <1 year, (2) hematological disease with bleeding tendency, (3) treatment with immunosuppressive agents, and (4) concomitant use of oral anticoagulation therapy.

All the clinical data, including coronary risk factors, major disease history, PCI procedures, and medications were collected from the patients' electrical medical records according to a pre-determined study protocol. The timing of aspirin discontinuation was obtained from the records of medications and the aspirin treatment duration was calculated accordingly. If possible, the reasons for aspirin discontinuation were also collected from the electronic medical records. For all included patients, the DAPT scores were calculated as previously reported (7). The DAPT score was calculated by assigning points according to the patients' characteristics, including age (0 for age <65 years, −1 for age 65–74 years, and −2 for age ≥75 years), smoking habit (1 for yes and 0 for no), diabetes mellitus (1 for yes and 0 for no), MI at presentation (1 for yes and 0 for no), prior PCI or MI (1 for yes and 0 for no), paclitaxel-eluting stent (1 for yes and 0 for no), stent diameter <3 mm (1 for yes and 0 for no), congestive heart failure or left ventricular ejection fraction <30% (2 for yes and 0 for no), and vein graft stent (2 for yes and 0 for no) (7). All the enrolled patients were divided into 2 groups according to their DAPT score: low (<2) or high (≥2) DAPT score. A high DAPT score (≥2) indicated that patients are at high ischemic risk and the ischemic benefits of prolonged DAPT therapy outweigh the bleeding risks. This study was conducted according to the principles expressed in the Declaration of Helsinki and was approved by the Institutional Medical Ethics Committee of National Cheng Kung University Hospital (IRB: A-ER-107-375). The principal investigators in all participating hospitals followed the study protocol strictly and the patients who did not meet the inclusion criteria were not included in this study.

### Follow-Up

The follow-up information was mainly obtained from the electronic medical records of the participating hospitals. The two major clinical outcomes of ischemic and bleeding endpoints were defined. The ischemic outcome is a composite endpoint of all-cause mortality, recurrent ACS or unplanned revascularization, and stroke within 12 months after the index PCI. All components of the composite endpoint were separately defined as secondary endpoints. All patients were followed up for at least 12 months after discharge or until one of the composite endpoints occurred. All these endpoint ischemic events were documented in the medical records of the patients and reported by the physicians who were responsible for patient follow-up. Recurrent ACS was defined as readmission to a hospital for management of new onset ST elevation MI, non-ST elevation MI, or unstable angina. Unplanned revascularization was defined as the first unexpected revascularization after discharge, including re-do PCI or a coronary artery bypass graft (CABG) after the index PCI due to new onset ischemic symptoms. Stroke, including ischemic or hemorrhagic stroke, was diagnosed by the occurrence of new-onset neurological symptoms and signs from neuroimaging studies. The bleeding outcome was defined as the occurrence of major bleeding as specified by the Bleeding Academic Research Consortium (BARC) type 3 and 5 bleedings (21).

### Statistical Analysis

Continuous variables were expressed as mean ± standard deviation and categorical variables were expressed as numbers and percentages. We used an unpaired Student's *t*-test for continuous variables and a chi-squared test for categorical variables to make comparisons between groups. The inverse probability of treatment weights (IPTW) method based on propensity scores was used to adjust for the imbalances in clinical characteristics between the groups, while preserving the sample size (22, 23). The propensity score was calculated according to the probability conditional at baseline characteristics, including age, sex, ST elevation MI status, diabetes mellitus, hypertension, hyperlipidemia, smoker, previous MI, previous PCI, previous CABG, previous ischemic stroke, previous hemorrhagic stroke, chronic kidney disease without dialysis, end stage renal disease with dialysis, heart failure, atrial fibrillation, peripheral artery disease, left ventricular ejection fraction, coronary angiography (CAG) finding, PCI procedure, location of treated lesion, stent, and medications.

In the IPTW model, we used the propensity score to generate patient-specific stabilized weights and to control for covariate imbalances. The propensity-score weight was calculated as the inverse of the propensity score for each patient. Comparisons of the clinical characteristics, CAG findings and PCI procedures, and medications between the groups were evaluated *via* absolute standardized mean difference (ASMD), which was calculated as the mean or proportion of a variable divided by the pooled estimate of the standard deviation of that variable. An ASMD >0.1 indicated a significant difference between the two groups. Cox proportional-hazard models were then adjusted for differences in the treatment groups using IPTWs derived from the propensity score, which was designated as the IPTW model. In the IPTW model after matching, the clinical characteristics with an ASMD >0.1 were put into the multivariate Cox proportional-hazards model for further adjustment. Because we already divided the groups by low and high DAPT scores, the criteria for the DAPT score were not in the multivariate model. Adjusted variables included body mass index >30, previous ischemic stroke, end-stage renal disease with dialysis, atrial fibrillation, CAG finding, PCI procedure, location of treated lesion, BMS, and statin use. Adjusted hazard ratios (HRs) and 95% CIs were calculated. We used the same Cox proportional hazards model to estimate the p values for interactions in the subgroup analysis. The SAS statistical package (version 9.4 for Windows; SAS Institute, Cary, NC, USA) was used for all analyses.

## Results

A total of 498 ACS patients (mean age 70.18 ± 12.84 years, men: 71.3%), who received PCI with stent implantation during hospitalization and survived to discharge, were included during the study period. There were 199 patients (40%) with low (<2) DAPT scores and 299 patients (60%) with high (≥2) DAPT scores. The mean time for aspirin treatment duration was similar between the low and high DAPT score groups (37.76 ± 52.67 vs. 41.90 ± 57.54 days, p = 0.471). Table 1 illustrates the reasons for premature discontinuation of aspirin. The most common reason for stopping aspirin was gastrointestinal bleeding (46.59%) with a similar percentage in both groups. Aspirin allergy and gastrointestinal upset were also common reasons for stopping aspirin. Aspirin allergy was significantly more common in the group with high DAPT score (*p* = 0.048), while gastrointestinal upset and discomfort were similar in both groups. Old age, anemia, or chronic use of oral non-steroidal anti-inflammatory drugs or steroids were other reasons for stopping aspirin. Unfortunately, the definite reason for stopping aspirin could not be identified in some patients from their medical records as this was a retrospective study.

**Table 1:** Reasons for premature discontinuation of aspirin.

**Reason**	**All**	**Low DAPT score**	**High DAPT score**	***p* value**
	***N =* 498 (%)**	***N =* 199 (%)**	***N =* 299 (%)**	
Gastrointestinal bleeding	232 (46.59)	95 (47.74)	137 (45.82)	0.742
Other sites bleeding	35 (7.03)	12 (6.03)	23 (7.69)	0.595
Aspirin allergy	53 (10.64)	14 (7.04)	39 (13.04)	0.048
Gastrointestinal upset or discomfort	48 (9.64)	18 (9.05)	30 (10.03)	0.833
Need surgery or thrombocytopenia	13 (2.61)	6 (3.02)	7 (2.34)	0.861
Other or unknown causes	117 (23.49)	54 (27.14)	63 (21.07)	0.145

*DAPT, dual antiplatelet therapy*.

Table 2 shows a comparison of baseline characteristics between the low and high DAPT score groups before and after matching. Since age, smoking, diabetes, prior PCI, prior MI, left ventricular ejection fraction <30%, and stent diameter <3 mm are the criteria included in the DAPT scoring system, it is natural to see a younger age and a higher proportion of these clinical characteristics in the high DAPT score group even after matching. The following characteristics, including body mass index, previous ischemic stroke, end stage renal disease with dialysis, atrial fibrillation, CAG finding, PCI procedure, location of treated lesion, use of stent, and use of statins, were also different between the groups and were further adjusted in the multivariate Cox proportional-hazards model for outcome evaluation.

**Table 2:** Baseline characteristics of patients with different DAPT scores.

**Characteristic**			**Inverse probability of treatment weighting**							
			**Before**					**After**		
	**All**		**Low DAPT score**		**High DAPT score**		**ASMD**	**Low DAPT score**	**High DAPT score**	**ASMD**
	***N =* 498**	**(%)**	***N =* 199**	**(%)**	***N =* 299**	**(%)**		**(*N =* pseudo data)**	**(*N =* pseudo data)**	
^*^Age	70.18 ± 12.84		75.72 ± 10.97		66.50 ± 12.68		0.777	76.86 ± 17.54	66.18 ± 15.44	0.647
Male	355	71.29	118	59.30	237	79.26	0.443	72.08	70.48	0.035
^*^BMI>30	43	8.63	7	3.52	36	12.04	0.322	3.79	8.49	0.197
STEMI	141	28.31	45	22.61	96	32.11	0.214	29.14	31.04	0.041
^*^Diabetes mellitus	271	54.42	66	33.17	205	68.56	0.757	24.57	70.30	1.030
Hypertension	376	75.50	149	74.87	227	75.92	0.024	77.98	76.23	0.042
Hyperlipidemia	273	54.82	106	53.27	167	55.85	0.052	54.38	55.35	0.020
^*^Smoker	146	29.32	17	8.54	129	43.14	0.860	7.48	44.66	0.935
^*^Previous MI	78	15.66	6	3.02	72	24.08	0.647	2.23	24.81	0.700
^*^Previous PCI	140	28.11	36	18.09	104	34.78	0.386	13.34	34.30	0.508
Previous CABG	16	3.21	5	2.51	11	3.68	0.067	2.39	3.98	0.090
^*^Previous ischemic stroke	76	15.26	31	15.58	45	15.05	0.015	9.77	13.01	0.102
Previous hemorrhagic stroke	3	0.60	2	1.01	1	0.33	0.082	0.65	0.48	0.024
CKD without dialysis	180	36.14	67	33.67	113	37.79	0.086	38.88	37.81	0.022
^*^ESRD with dialysis	68	13.65	24	12.06	44	14.72	0.078	9.38	12.89	0.112
Heart failure	168	33.73	17	8.54	151	50.50	1.036	35.34	34.70	0.013
^*^Atrial fibrillation	66	13.25	22	11.06	44	14.72	0.109	8.18	15.24	0.221
Peripheral artery disease	32	6.43	15	7.54	17	5.69	0.075	5.77	6.13	0.015
Bleeding history	158	31.73	70	35.18	88	29.43	0.123	28.11	31.15	0.066
^*^LVEF	57.17 ± 14.53		62.55 ± 12.01		53.59 ± 14.97		0.660	58.49 ± 21.21	55.92 ± 17.98	0.130
Hb (g/dL)	12.14 ± 2.81		11.93 ± 2.47		12.28 ± 3.01		0.126	11.94 ± 4.31	12.06 ± 3.86	0.030
WBC	9,822.24 ± 4,308.33		9,214.77 ± 4,099.82		10,226.54 ± 4,402.09		0.238	9,275.96 ± 6,468.48	9,779.31 ± 5,415.31	0.084
^*^CAG finding							0.050			0.188
1-vessel disease	123	24.70	48	24.12	75	25.08	0.022	36.99	28.42	0.184
2-vessel disease	141	28.31	59	29.65	82	27.42	0.049	26.67	28.56	0.042
3-vessel disease	234	46.99	92	46.23	142	47.49	0.025	36.35	43.02	0.137
^*^PCI procedure							0.018			0.152
Single lesion intervention	278	55.82	110	55.28	168	56.19		49.08	58.45	
Multiple lesions intervention	220	44.18	89	44.72	131	43.81		49.08	41.55	
^*^Location of lesion treated										
LM	38	7.63	17	8.54	21	7.02	0.057	4.83	6.53	0.073
LAD	319	64.06	130	65.33	189	63.21	0.044	55.26	63.18	0.162
LCX	194	38.96	78	39.20	116	38.80	0.008	31.83	38.42	0.138
RCA	234	46.99	94	47.24	140	46.82	0.008	52.45	44.55	0.159
SVG	2	0.40	0	0.00	2	0.67	0.116	-	0.90	
^*^Stent										
Bare metal stent	214	42.97	80	40.20	134	44.82	0.094	35.84	43.62	0.159
Everolimus-eluting stent	93	18.67	35	17.59	58	19.40	0.047	17.16	19.61	0.063
Zotarolimus-eluting stent	99	19.88	43	21.61	56	18.73	0.072	17.40	19.26	0.048
Biolimus-eluting stent	26	5.22	14	7.04	12	4.01	0.133	7.07	4.75	0.098
Sirolimus-eluting stent	65	13.05	31	15.58	34	11.37	0.123	17.55	12.00	0.157
Stent <3 mm	200	40.16	60	30.15	140	46.82	0.348	20.12	52.30	0.711
Medications										
Clopidogrel	271	54.42	118	59.30	153	51.17	0.164	51.15	46.26	0.098
Ticagrelor	227	45.58	81	40.70	146	48.83	0.164	48.85	53.74	0.098
Beta blocker	367	73.69	133	66.83	234	78.26	0.258	75.79	73.35	0.056
RAS inhibitor	283	56.83	105	52.76	178	59.53	0.137	57.64	56.88	0.015
^*^Statin	405	81.33	153	76.88	252	84.28	0.188	75.87	82.19	0.156
PPI use	203	40.76	96	48.24	107	35.79	0.254	39.63	40.53	0.018

*BMI, body mass index; CABG, coronary artery bypass graft; CAG, coronary angiography; CKD, chronic kidney disease; DAPT, dual antiplatelet therapy; ESRD, end stage renal disease; LAD, left anterior descending artery, LCX, left circumflex artery; LM, left main artery; LVEF, left ventricular ejection fraction; MI, myocardial infarction; PCI, percutaneous coronary intervention; PPI, proton pump inhibitor; RAS, renin angiotensin system; RCA, right coronary artery; STEMI, ST-segment elevation myocardial infarction; SVG, saphenous vein graft; ASMD, absolute standardized difference*.

* *ASMD >0.1 between the groups*.

All patients were followed up by the physicians who enrolled the patients. The mean duration of follow-up was 341.68 ± 67.58 and 330.97 ± 86.62 days in the low and high DAPT score groups, respectively (*p* = 0.123). Table 3 shows the clinical outcomes during the 12-month follow-up after the index PCI. The composite ischemic outcome occurred in 11.56% of the low and 14.38% of the high DAPT score group and there was no significant difference between the groups after multivariate adjustment (adjusted HR: 1.169, 95% CI: 0.832–1.643). For the secondary endpoint, there was also no significant difference in stroke and all-cause death between the two groups. However, the risk of recurrent ACS or unplanned revascularization was significantly higher in the high DAPT score group (adjusted HR: 1.900, 95% CI: 1.095–3.295) compared with the low DAPT score group. BARC 3 and 5 bleeding occurred in 3.02% of the low and 4.01% of the high DAPT score group. There was no significant difference in BARC 3 and 5 bleeding (adjusted HR: 1.206, 95% CI: 0.623–2.335) between the two groups.

**Table 3:** Clinical outcomes at 12-months follow-up.

**Outcome**	**All**	**Low DAPT score**	**High DAPT score**	**Crude HR**	***p* value**	**Adjusted HR**	***p* value**
	***N =* 498**	***N =* 199 (Ref)**	***N =* 299**	**(95% CI)**		**(95% CI)**	
Primary composite endpoint	66 (13.25)	23 (11.56)	43 (14.38)	0.792 (0.579–1.082)	0.143	1.169 (0.832–1.643)	0.367
Secondary endpoint							
Recurrent ACS or unplanned revascularization	41 (8.23)	12 (6.03)	29 (9.70)	1.965 (1.145–3.372)	0.014	1.900 (1.095–3.295)	0.022
Stroke	1 (0.20)	0	1 (0.33)	-		-	
All-cause death	24 (4.82)	11 (5.53)	13 (4.35)	0.426 (0.277–0.654)	<0.001	0.758 (0.465–1.237)	0.268
BARC 3 or 5 bleeding	18 (3.61)	6 (3.02)	12 (4.01)	1.341 (0.709–2.539)	0.367	1.206 (0.623–2.335)	0.578

*ACS, acute coronary syndrome; BARC, Bleeding Academic Research Consortium; DAPT, dual antiplatelet therapy*.

*Adjusted variables included body mass index >30, previous ischemic stroke, end stage renal disease with dialysis, atrial fibrillation, coronary angiography finding, percutaneous coronary intervention procedure, location of treated lesion, bare metal stent, and statin*.

**Figure 1:**
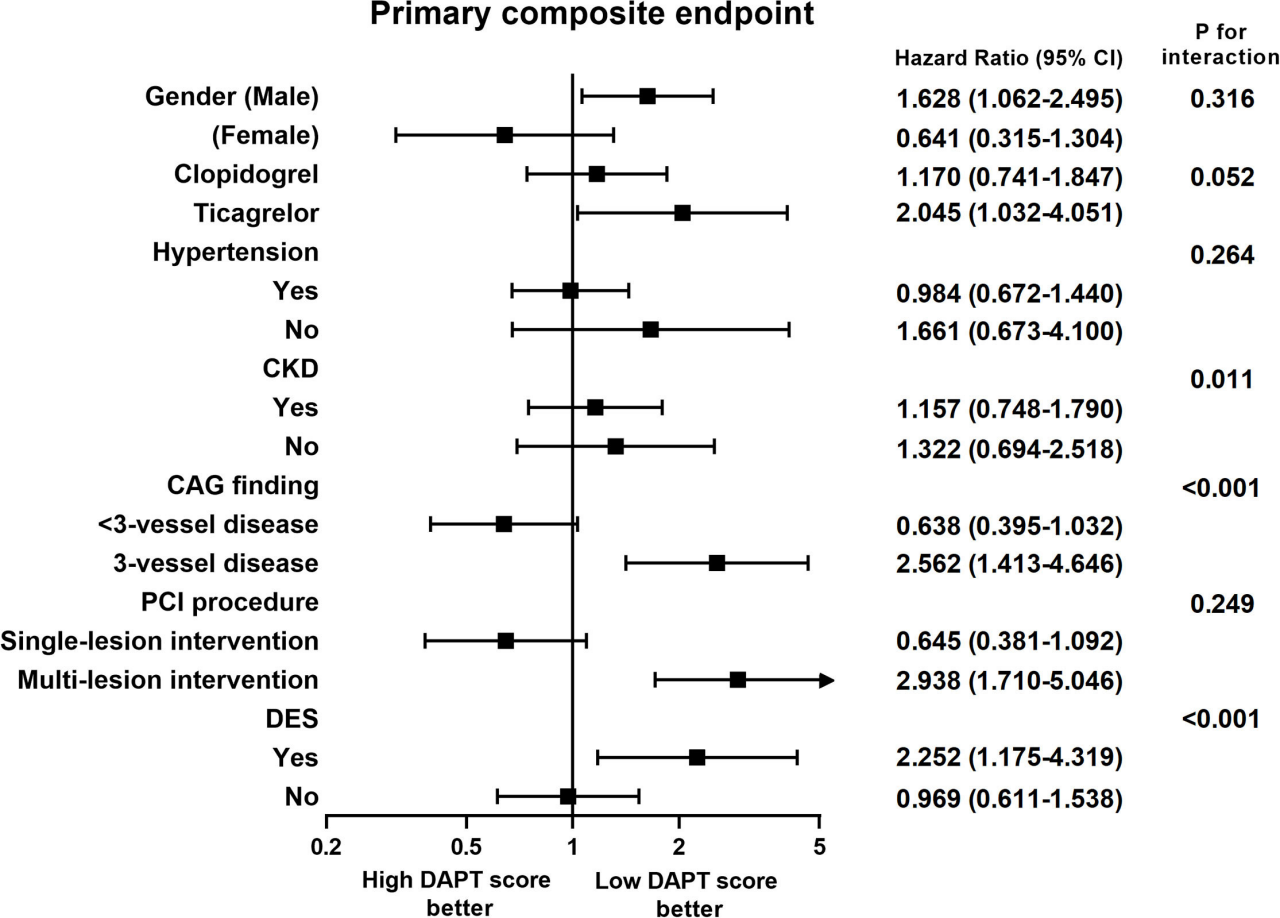
Subgroup analysis of the effect of different dual antiplatelet therapy scores on primary composite endpoints. CAG, coronary angiography; CKD, chronic kidney disease; DES, drug-eluting stent; PCI, percutaneous coronary intervention.

Figure 1 shows the subgroup analysis results regarding sex, clopidogrel or ticagrelor, hypertension, chronic kidney disease, 3-vessel disease, single or multiple-lesion intervention, and DES between the two groups. The criteria in the DAPT score, such as age, smoking, diabetes, prior PCI, prior MI, left ventricular ejection fraction <30%, and stent diameter <3 mm, were not used for the subgroup analysis. In the subgroup analysis, patients with high DAPT score had a higher risk of primary composite endpoint in the subgroups of chronic kidney disease, 3-vessel disease, and DES (*p* for interaction <0.05). There was a borderline interaction between those treated with ticagrelor or clopidogrel (*p* for interaction = 0.052).

## Discussion

In this study, we assessed the association between the DAPT score and the clinical outcomes in ACS patients receiving P2Y12 inhibitor monotherapy after index PCI. We found that the DAPT score was useful for determining the ischemic risk in these patients. Although previous clinical trials found that the efficacy of P2Y12 inhibitor monotherapy for preventing ischemic events was comparable to standard 12-month DAPT, the present study demonstrated that patients with high DAPT score still had a significantly higher rate of recurrent ACS or unplanned revascularization compared with those with low DAPT score.

After its development in the DAPT trial, the DAPT score has been extensively investigated with regard to its ability to stratify ischemic risk in a wide variety of patient groups who have received PCI (8–11). Most of the studies confirmed that patients with a high DAPT score have a higher incidence of ischemic events when compared with patients with a low DAPT score. For the strategy of very short DAPT (1–3 months) followed by P2Y12 inhibitor monotherapy, the influence of the DAPT score remains unclear. GLOBAL LEADERS trial is a randomized study comparing 1 year of DAPT therapy (aspirin plus clopidogrel or ticagrelor) followed by 1 year of aspirin monotherapy with 1 month of DAPT therapy (aspirin plus ticagrelor) followed by 23 months of ticagrelor, among patients undergoing PCI for stable CAD or ACS (12). A recent study analyzed the clinical outcomes from the second year follow up with aspirin or ticagrelor monotherapy in the GLOBAL LEADERS trial. It demonstrated that patients with high DAPT score had a significantly higher rate of the composites of MI or stent thrombosis (0.70% vs. 1.55%, *p* < 0.0001) and a similar rate of BARC type 3 or 5 bleeding (24). The authors concluded that the DAPT score can stratify ischemic risk but not bleeding risk in a contemporary PCI population during the second year.

Our study only included ACS patients receiving P2Y12 inhibitor monotherapy (ticagrelor or clopidogrel), and we observed the first year outcomes after PCI. We initially hypothesized that a high DAPT score would predict higher composite ischemic events, but the results were not as expected. The potential reasons for this are the limited follow-up time (12 months) and the small case number. However, we did find that the risk of recurrent ACS or unplanned revascularization was significantly higher in the high DAPT score group. In addition, our study also found that the DAPT score could not stratify bleeding risk in ACS patients undergoing PCI, which was similar to the results of the GLOBAL LEADERS trial (24). Subgroup analysis revealed that there were more ischemic events in the high DAPT score group with 3-vessel disease, and coronary anatomy complexity is a known risk factor for MACEs after PCI (25, 26).

Our study results indicate that early discontinuation of aspirin with P2Y12 inhibitor monotherapy should be the last resort for ACS patients with 3-vessel disease and a high DAPT score because the high ischemic risk is still a major concern. Overall, the patients receiving BMS deployment had higher ischemic events than those receiving DES deployment (recurrent ACS or unplanned revascularization: BMS 11.21 vs. DES 6.72%). However, in the subgroup analysis, we found that patients receiving P2Y12 inhibitor monotherapy with ticagrelor and DES deployment, had higher primary composite endpoints in the high DAPT score group. Probable reasons to explain this result are that the choice of P2Y12 inhibitor and stent in real-world practice are based on the clinician's experience and the local insurance system. Ticagrelor is more expensive than clopidogrel. A previous real-world observation study of ACS in Taiwan demonstrated that ticagrelor offers a better protective effect against ischemic events when compared with clopidogrel (27). However, BMS is still commonly used in Taiwan because the Taiwan National Health Insurance only reimburses the price of BMS. Patients using DES have to pay the price difference, which is around US$1,500 to US$2,000 for one DES (28). Therefore, it is likely that physicians in Taiwan are prone to choosing ticagrelor and DES for ACS patients with a higher ischemic risk and recurrent events.

To the best of our knowledge, this is the first study to assess the ability of the DAPT score to stratify ischemic and bleeding risk in ACS patients with P2Y12 inhibitor monotherapy undergoing PCI in Asia. However, our study did have several limitations. First, our study was a retrospective, nonrandomized, observational study. The unadjusted confounding factors were unavoidable, even though a propensity score-matched analysis was used to compensate for this. Second, the case number was relatively small in our study, which may have caused selection bias of the included patients. Standard 12-month DAPT is still the recommended therapy for ACS patients who undergo PCI in Taiwan (29). Therefore, it is difficult to collect large case numbers of patients with only P2Y12 inhibitor monotherapy from real-world practice. Third, there was only a 12-month follow-up after PCI. A longer follow-up duration may be necessary to determine the definite association of the DAPT score with the clinical outcomes. Fourth, although the guidelines recommend new-generation P2Y12 inhibitors and DES for ACS patients undergoing PCI, BMS and clopidogrel are still commonly used in Taiwan for various reasons. Our study results may be different if all patients were treated with ticagrelor and DES. Fifth, we did not know the exact cause of death in all 24 patients with mortality. Some patients died from sepsis/pneumonia, cancer, and respiratory failure, but not cardiovascular causes. Finally, our results may not be applicable to non-Asian patients.

In conclusion, ACS patients receiving early P2Y12 monotherapy with high DAPT score had a higher risk of recurrent ACS or unplanned revascularization compared with those with low DAPT score. The risk of major bleeding was similar between those with low and high DAPT scores. This study suggests that the DAPT score is validated for predicting cardiovascular events in ACS patients undergoing PCI with short DAPT treatment followed by P2Y12 inhibitor monotherapy.

## Data Availability Statement

The original contributions presented in the study are included in the article/supplementary material, further inquiries can be directed to the corresponding authors.

## Ethics Statement

The studies involving human participants were reviewed and approved by the Institutional Medical Ethics Committee of National Cheng Kung University Hospital (IRB: A-ER-107-375). Written informed consent was not required for this study, in accordance with the local legislation and institutional requirements.

## Author Contributions

M-YH, W-HF, C-FT, C-HS, S-WH, C-WC, H-IY, C-PC, W-CH, C-CF, I-CH, and Y-HL were responsible for the conceptualization. Data curation was done by M-YH, P-WC, W-HF, C-FT, C-HS, S-WH, C-WC, H-IY, C-PC, W-CH, C-CF, I-CH, and Y-HL. M-YH, P-WC, W-HF, C-HS, S-WH, C-WC, H-IY, C-PC, W-CH, C-CF, I-CH, and Y-HL performed the formal analysis. Funding acquisition was taken care of by W-HF and I-CH. M-YH, P-WC, W-HF, C-HS, H-WL, and Y-HL carried out the investigation. P-WC, W-HF, H-WL, and S-HL took care of the methodology. Project administration was the responsibility of C-HS, P-WC, W-HF, and Y-HL. H-WL and S-HL handled the software. C-HS, C-FT, S-HL, I-CH, and Y-HL supervised the study. C-FT, H-WL, S-HL, and I-CH performed the validation. C-FT, S-HL, and I-CH visualized the study. Writing of the original draft was done by S-WH, P-WC, C-HS, and Y-HL. C-HS, S-WH, Y-HL, and P-WC reviewed and edited the study. All authors contributed to the article and approved the submitted version.

## Funding

The authors gratefully acknowledge the support of Kaohsiung Municipal Ta-Tung Hospital (kmtth-109-009).

## Conflict of Interest

The authors declare that the research was conducted in the absence of any commercial or financial relationships that could be construed as a potential conflict of interest.

## Publisher's Note

All claims expressed in this article are solely those of the authors and do not necessarily represent those of their affiliated organizations, or those of the publisher, the editors and the reviewers. Any product that may be evaluated in this article, or claim that may be made by its manufacturer, is not guaranteed or endorsed by the publisher.
